# Lichen Endozoochory by Snails

**DOI:** 10.1371/journal.pone.0018770

**Published:** 2011-04-13

**Authors:** Steffen Boch, Daniel Prati, Silke Werth, Jörg Rüetschi, Markus Fischer

**Affiliations:** 1 Institute of Plant Sciences, Botanical Garden, University of Bern, Bern, Switzerland; 2 Oeschger Center, University of Bern, Bern, Switzerland; 3 Biodiversity and Conservation Biology, Swiss Federal Research Institute WSL, Birmensdorf, Switzerland; 4 Hinterkappelen, Switzerland; University of Hull, United Kingdom

## Abstract

Endozoochory plays a prominent role for the dispersal of seed plants. However, for most other plant taxa it is not known whether this mode of dispersal occurs at all. Among those other taxa, lichens as symbiotic associations of algae and fungi are peculiar as their successful dispersal requires movement of propagules that leaves the symbiosis functional. However, the potential for endozoochorous dispersal of lichen fragments has been completely overlooked. We fed sterile thalli of two foliose lichen species (*Lobaria pulmonaria* and *Physcia adscendens*) differing in habitat and air-quality requirements to nine snail species common in temperate Europe. We demonstrated morphologically that *L. pulmonaria* regenerated from 29.0% of all 379 fecal pellets, whereas *P. adscendens* regenerated from 40.9% of all 433 fecal pellets, showing that lichen fragments survived gut passage of all snail species. Moreover, molecular analysis of regenerated lichens confirmed the species identity for a subset of samples. Regeneration rates were higher for the generalist lichen species *P. adscendens* than for the specialist lichen species *L. pulmonaria*. Furthermore, lichen regeneration rates varied among snail species with higher rates after gut passage of heavier snail species. We suggest that gastropods generally grazing on lichen communities are important, but so far completely overlooked, as vectors for lichen dispersal. This opens new ecological perspectives and questions the traditional view of an entirely antagonistic relationship between gastropods and lichens.

## Introduction

Dispersal is among the most important mechanisms shaping biodiversity. In contrast to mobile animals, sessile plants depend on dispersal of propagules or plant fragments. Endozoochory plays a prominent role for the dispersal of seed plants, and seed dispersal vectors are well known [1]–[3]. However, whether endozoochory occurs is not known for most other plant taxa. Among those, lichens as symbiotic associations of algae and fungi are peculiar as their successful dispersal requires that the symbiosis remains functional.

Lichens occur in essentially all terrestrial and some aquatic habitats [4]. They reproduce vegetatively by symbiotic propagules, thallus fragments, or by producing fungal spores. Lichen dispersal via fungal spores, however, involves reconstituting the symbiosis with algae and is not further considered here. Lichen propagules or fragments can be dispersed by wind, water, or exozoochory. Lichens are consumed by various animals including gastropods and arthropods [5]. Lichen feeders may have a strong effect on lichen abundance and community composition [6], [7]. E.g., Asplund & Gauslaa [8] showed that lichen-feeding snails limit establishment and survival of young *Lobaria pulmonaria* thalli. On the other hand, independent of each other, both photobionts and fungal spores can survive the gut passage of lichen feeders [9], [10] which may be beneficial for lichens, if it leads to successful new constitution of the lichen symbiosis. However, survival of animal-gut passage by whole lichen fragments, and, thus, the potential for endozoochorous lichen dispersal, which would be a much simpler and more direct way of lichen dispersal, has been completely overlooked.

Therefore, also any information is lacking about differences between lichen species and between lichen feeders in lichen survival of the gut passage. Possibly, such survival might be higher for more generalized lichen species and for larger lichen feeders ingesting larger lichen fragments.

Increasingly, DNA-based methods allow addressing taxonomic, phylogenetic, and population-genetic questions in lichens [11]. Recently, species-specific molecular markers have become available which can support species identification traditionally based on morphology and secondary chemistry.

In this study, we address lichen dispersal by snails because many snails feed almost exclusively on lichens. In particular, we tested experimentally (1) whether vegetative lichen propagules can survive and regenerate after the passage through snail guts, i.e. whether lichen endozoochory is possible, (2) whether regeneration rates differ between a specialist and a generalist lichen species, and (3) whether snail species differ in their efficiency to disperse lichens and whether this depends on their body mass.

## Results

**Figure 1:**
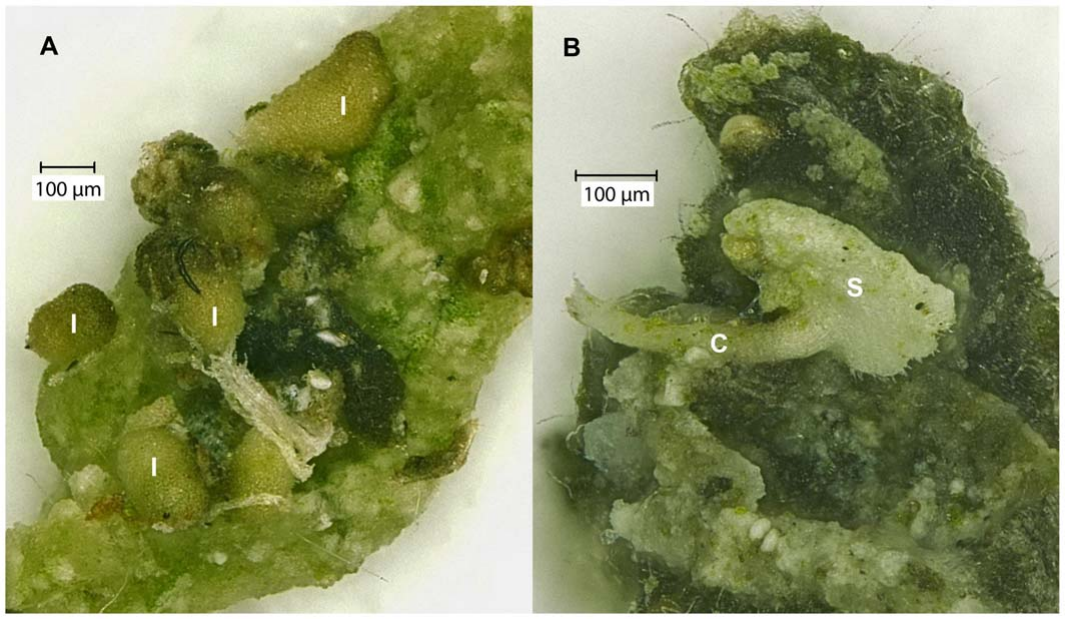
Microscope images of lichen species regenerated from snail feces. A) I: isidioid soredia of *L. pulmonaria*. B) S: squamule (shell-formed thallus piece), and C: cilium (thread-like appendage) of *P. adscendens*.

*Lobaria pulmonaria* regenerated from 29.0% of all 379 fecal pellets, whereas *P. adscendens* regenerated from 40.9% of all 433 fecal pellets, indicating that lichen fragments passed the snail guts without being digested and developed into juvenile lichens (Figure 1). The differences in regeneration rate between the two species were significant (Table 1). For *L. pulmonaria*, we mainly found isidioid soredia (in 83 of 379 fecal pellets), sometimes small squamules (in 37 of 379 fecal pellets) and rarely also hyphae (18 of 379 fecal pellets). For *P. adscendens*, we mainly observed squamules (in 168 out of 433 fecal pellets) or cilia (in 170 of 433 fecal pellets), and very rarely small soredia clusters (in 2 of 433 fecal pellets).

**Table 1:** Results of GLM analysis (with quasi-binomial error structure) of differences between the two lichen species (generalist versus specialist lichen species as fixed factor) and among the nine snail species (as random factor) in lichen regeneration rates.

	Regeneration rate of lichens			
Source of variation	df	Deviance	*F*	*p*
Lichen species	1	12.51	18.256	0.003
Snail species	8	99.15	18.080	<0.001
*Snail mass*	*1*	*46.41*	*6.160*	*0.042*
*Residual variation among snail species*	*7*	*52.74*	*10.992*	*0.002*
Lichen species×snail species	8	5.75	0.386	0.927
Residuals	188	401.4		

The model also included snail body mass as linear contrast and the interaction between lichen and snail species.

**Figure 2:**
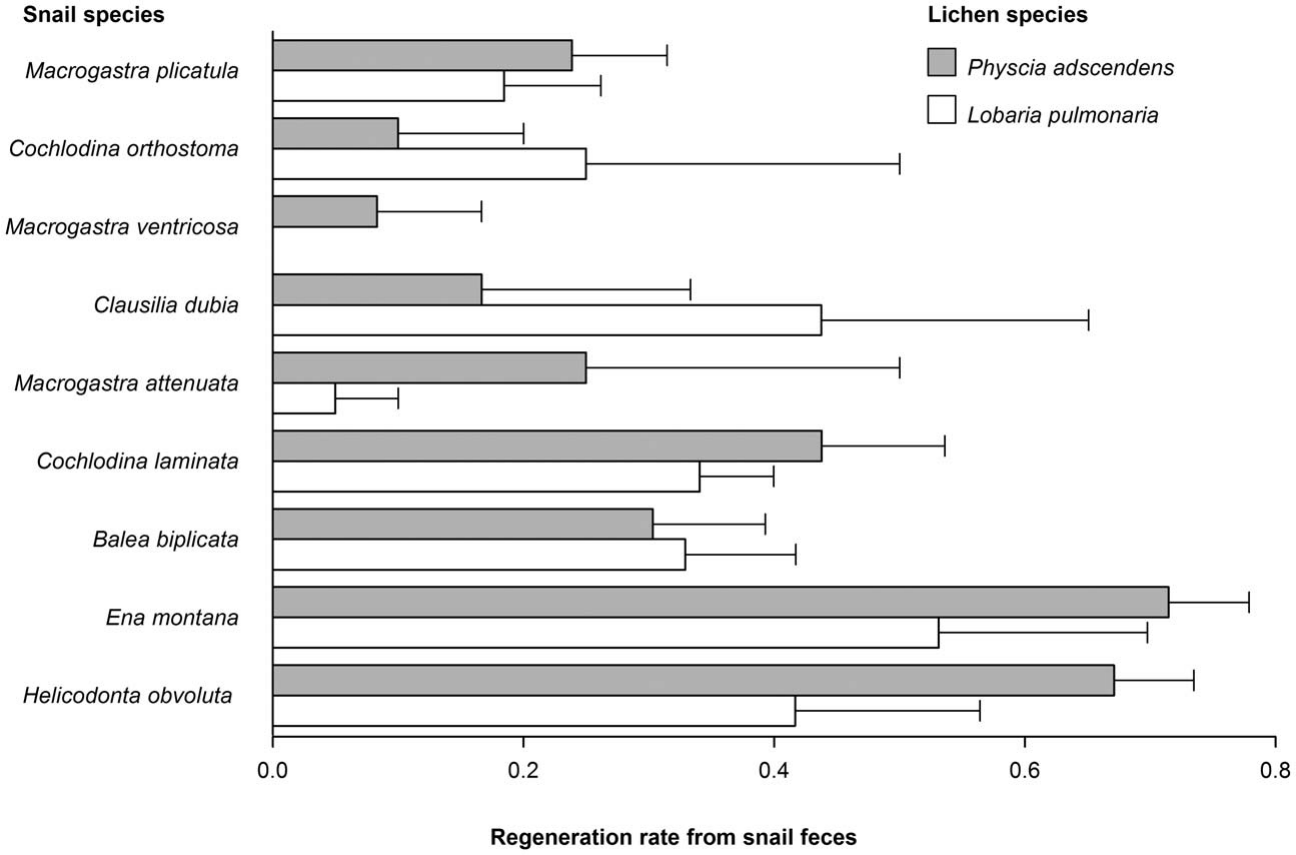
Variation in regeneration rate of lichen species from fecal pellets of snail species (means +s.e.m). Snail species are sorted from smallest to largest body mass.

Furthermore, lichen regeneration rate varied among snail species (Table 1; Figure 2). Regeneration rates of lichens were higher after gut passage of heavier snail species (Table 1). Significant variation among snail species remained after accounting for snail mass (Table 1). Effects of snail and lichen species were independent of each other (non-significant snail species - by - lichen species interaction, Table 1).

Molecular analysis fully confirmed morphological determination of species identity for the 16 analysed regenerated specimens of *L. pulmonaria* and the 6 of *P. adscendens*. The eight fungal microsatellite markers amplified with all 16 samples representing regenerated isidioid soredia of *L. pulmonaria*. All seven algal markers amplified for 11 of the samples and three to six algal markers amplified for another five samples. Together these results indicate that the analyzed samples were indeed regeneration structures of *L. pulmonaria* which contained both the fungus and its photobiont. All sequences obtained from samples of *P. adscendens* blasted to sequences of that same species. For two further samples of *P. adscendens*, DNA extraction had most likely failed, as we did not obtain a PCR product with the fungus-specific PCR primers. None of the negative controls showed amplification products.

Overall, we conclude that snails can generally act as endozoochorous dispersers of the two lichen species, with higher regeneration rates for the generalist lichen, and with some, especially heavier, snail species allowing higher regeneration rates after gut passage than others.

## Discussion

Vegetative lichen propagules and fragments regenerated from snail feces, implying that endozoochorous lichen dispersal is possible. Previously it was shown that fungal spores or single algal cells can survive animal gut passage [9], [10], but complete lichen dispersal in this way would require successful reconstitution of the symbiosis after gut passage. In our case, the symbiotic life-form as a whole survived and thus can be dispersed endozoochorously, which very likely is a more efficient mode of lichen dispersal than via spores. Population ecological studies on *L. pulmonaria* emphasized the role of establishment limitation for lichens [12] because successful establishment of lichen propagules requires placement on appropriate substrate [5]. We suggest that endozoochory by snails may contribute considerably to overcoming this limitation by placing and, due to sticky feces, attaching vegetative fragments on appropriate substrate. Snail feces may even foster lichen growth with its nutrients. Especially for lichen species with heavy symbiotic propagules, such as *L. pulmonaria*, these effects may be advantageous by increasing local population sizes.

Larger snails may be especially important dispersers because their fecal pellets rather contained undigested thallus fragments than the ones of smaller snail species (Figure 2). Larger snails are likely to ingest larger lichen fragments, which may explain why gut passage of lichen fragments through larger snail species yielded higher regeneration rates. At the same time this is likely to involve larger fecal pellets than for smaller snail species. The size and quality of ingested lichen material and the size and quality of fecal pellets of larger and smaller snails and their importance for lichen dispersal will be interesting next steps in investigating lichen dispersal by snails.

Our results are consistent across several common snail species. It remains more open, whether endozoochorous lichen dispersal can be generalized among lichen species. We used two foliose lichen species differing in abundance and demands regarding habitat and air quality, and found higher regeneration rates for the generalist lichen species *Physcia adscendens* than for the specialist lichen species *Lobaria pulmonaria*. Lichen growth form may be relevant for regeneration of thallus material after gut passage. So far, it appears that only fruticose species [13] or foliose, squamulose, and lobate crustose species [5] are able to regenerate from thallus fragments at all. Accordingly, a previous study of field-sampled feces failed to show endozoochorus lichen dispersal of several tropical lichen feeders, including snails, which fed mainly on crustose foliicolus lichen species [6]. However, as vegetative propagation by soralia or isidia is common also in crustose lichen species, the possibility of endozoochory cannot generally be excluded for those lichens.

Our experiment demonstrated that endozoochorous dispersal of lichen fragments by gastropods is possible. It implies that, despite losses due to gastropod herbivory, lichen thalli might be proliferated by gastropods due to fragmentation into several pieces. Thus, endozoochorous lichen dispersal by gastropods may even increase lichen population growth, and thus constitute a gastropod-fungus-algae mutualism between organisms of three kingdoms.

The importance of lichen endozoochory relative to dispersal by water or wind remains open. In temperate and boreal regions, gastropods feed only in warmer seasons, whereas wind and water dispersal can occur throughout the year. However, lichen feeding by gastropods is expected to increase with climate change due to milder winters and higher annual precipitation [14], which may further enhance the importance of endozoochorous lichen dispersal by gastropods.

Endozoochorous lichen dispersal by gastropods probably plays a more minor role for long-distance than for short-distance dispersal. Nevertheless, slugs may disperse plant seeds at least up to 15 m [3], and the same slugs frequently feed on lichens [15]. Moreover, as gastropods can themselves be dispersed over long distances by wind or animals [16], [17], lichens “stowing away” in gastropods may be important for long-distance lichen dispersal.

In conclusion, the traditional view of an entirely antagonistic herbivorous relationship between gastropods and lichens [5], [8], [18] must be challenged. Given that gastropod grazing on lichen communities is very common [5], our findings imply that fragmentation, and thus proliferation, and dispersal of lichen thalli by gastropods may be an important, but so far completely overlooked, mode of lichen dispersal and expansion into suitable habitats. Possibly gastropods not only profit directly from feeding on lichens, but also indirectly from enhancing lichen population growth by endozoochorous dispersal. In any case, lichen endozoochory by gastropods may well constitute a mutualistic relationship between partners from three different kingdoms.

## Materials and Methods

### Lichen species

Sterile thalli of two foliose lichen species were collected from bark. *Physcia adscendens* (Fr.) H. Olivier, a frequent generalist lichen species throughout Central Europe growing on various nutrient-enriched substrates, was collected on *Euonymus europaea* L. in Switzerland (46°57′N 7°26′E). *Lobaria pulmonaria* (L.) Hoffm. was collected on *Salix caprea* L. in Norway (64°26′N 11°47′E). *L. pulmonaria* is vulnerable or endangered in most of Central Europe because of its sensitivity to air pollution and preference for old-growth forests. The species is limited by its very low establishment rates [12]. It is widely used as a model species in ecophysiological, ecological and conservation biological research [19].

### Snail species

We collected adults of nine snail species commonly occurring on rocks and tree bark in temperate European forests: *Balea biplicata* (*N* = 8; 148.6±5.7 mg), *Clausilia dubia* (*N* = 1; 122.5 mg), *Cochlodina laminata* (*N* = 13; 137.9±5.0 mg), *Cochlodina orthostoma* (*N* = 2; 74.7±5.3 mg), *Ena montana* (*N* = 20; 208.5±17.3 mg), *Helicodonta obvoluta* (*N* = 20; 414.2±6.7 mg), *Macrogastra attenuata* (*N* = 1; 128.7 mg), *Macrogastra plicatula* (*N* = 14; 66.0±1.6 mg), *Macrogastra ventricosa* (*N* = 1; 111.9 mg). They were all sampled from bark in a beech forest in Germany (48°2′N 9°26′E; [20]) and returned to the sampling site after the experiment. Snail nomenclature follows Turner et al. [21].

### Feeding experiment and lichen cultivation

Snails were individually kept in Petri dishes for 48 hours and fed with tissue-paper to assure defecation. Then, we fed moistened lichen thalli (0.2 g dry mass) randomly assigned to each snail for 24 hours (20°C; 12/12 h light/dark cycle). Individual snails were used in two to three trials and were fed with tissue-paper for 48 hours between trials such that defecation was independent across trials. After each trial we cleaned snails with water and shells with alcohol to avoid secondary contamination with lichen propagules and transferred them for 24 hours into new Petri dishes with tissue-paper for defecation. During this time, all fecal pellets were collected and put on parafilm stripes placed on filter paper in one separate Petri dish per snail individual to exclude contact of fecal pellets tested for regeneration with lichen material. We incubated fecal pellets from 1 September to 23 October 2009 (17/15°C; 16/8 h light/dark cycle). We moistened the filter paper weekly with deionized water to assure a wetting-drying cycle and to minimize potential growth of non-lichen fungi or bacteria. In total, we cultivated 433 fecal pellets from snails that had fed on *P. adscendens* in 111 Petri dishes and 379 fecal pellets from snails that had fed on *L. pulmonaria* in 95 Petri dishes.

### Morphological identification of lichen growth

We inspected all single pellets of feces of each individual snail with a dissecting microscope and recorded the number of fecal pellets per Petri dish from which lichen structures had regenerated.

### Molecular analysis

We dissected regenerated lichen structures from putative samples of *L. pulmonaria* (*N* = 17) and *P. adscendens* (*N* = 7), ground them using stainless steel beads in a mixer mill (Retsch MM 2000, Haan, Germany), and extracted DNA of individual isidioid soredia and squamules (according to Werth et al. [22]) using the Sigma GenElute Plant Genomic DNA Miniprep kit (Sigma-Aldrich, St. Louis, MO, USA) following the manufacturer's instructions Two negative controls were included in the DNA extraction.

For *L. pulmonaria*, we performed molecular species identifications using species-specific RealTime PCR based on the fungal ITS region and based on eight genus-specific fungal microsatellite loci [23], [24]. We also analyzed seven photobiont microsatellite loci (LPh1–LPh7) following Dal Grande et al. [25]. We ran fragment analyses on an automated capillary sequencer (3130 Genetic Analyzer, Applied Biosystems, Rotkreuz, Switzerland), and typed alleles using an internal size standard (LIZ500, Applied Biosystems, Rotkreuz, Switzerland). Samples were genotyped using GeneMapper version 3.7 (Applied Biosystems, Rotkreuz, Switzerland).

For *P. adscendens*, we sequenced the ITS region using the ascomycete-specific primer ITS1F [26] and the universal reverse primer ITS4 [27]. Each 25 µL reaction contained 1× JumpStart REDTaq Ready Mix (Sigma-Aldrich, St. Louis, Missouri, USA), 100 nM of each primer, and 1 µL DNA extract (ca. 0.1 ng). The cycling conditions of the PCR included an initial denaturation at 94°C for 2 min, then 34 cycles of 30 s at 94°C, 30 s at 50°C, 30 s at 72°C, followed by final extension of 10 min at 72°C. We performed cycle sequencing and reaction clean-up following Werth and Sork [28] and edited sequences using the program Sequence Scanner, version 1.0 (Applied Biosystems, Foster City, CA, USA). Finally, we performed BLAST searches [29] to identify the species corresponding to the DNA sequences (GenBank accessions HM246686–HM246691).

### Statistical analysis

For each of the 206 Petri dishes, we determined regeneration rate as proportion of fecal pellets with regenerated lichen structures. We tested differences in regeneration rate between lichen species (generalist versus specialist lichen species as fixed factor) and among snail species (as random factor) with a generalized linear model with quasi-bionominal link function because of overdispersion. We included mean body mass, as covariate tested against the snail species level, and the interaction between lichen and snail species. Data were analyzed using *R*, Version 2.6.1 [30].
